# Socio-emotional and motor engagement during musical activities in older adults with major neurocognitive impairment

**DOI:** 10.1038/s41598-021-94686-4

**Published:** 2021-07-27

**Authors:** Lise Hobeika, Matthieu Ghilain, Loris Schiaratura, Micheline Lesaffre, Dominique Huvent-Grelle, François Puisieux, Séverine Samson

**Affiliations:** 1 Université de Lille, ULR 4072 – PSITEC – Psychologie: Interactions, Temps, Emotions, Cognition, 59000 Lille, France; 2 Sciences et Technologies de la Musique et du Son, IRCAM, CNRS, Sorbonne Université, 75004 Paris, France; 3 IPEM, Department of Art History, Musicology and Theatre Studies, Ghent University, 9000 Ghent, Belgium; 4 Hôpital Gériatrique les Bateliers, Pôle de Gérontologie, CHU Lille, 23 rue des Bateliers, 59037 Lille, France; 5 AP-HP, GHU Pitié-Salpêtrière-Charles Foix, 75013 Paris, France

**Keywords:** Dementia, Social neuroscience, Cognitive ageing, Emotion, Social behaviour, Human behaviour

## Abstract

Although music therapy may engender clinical benefits in patients with neurodegenerative disease, the impacts of social and musical factors of such activities on socio-emotional and motor engagements are poorly understood. To address this issue, non-verbal behaviors of 97 patients with or without major cognitive impairment (CI) were assessed when listening to music or a metronome in front of a musician who was present physically (live) or virtually (video). Socio-emotional engagement was quantified as emotional facial expression production and gaze direction. Motor engagement was quantified as overall body motion and the production of rhythmic movements. In both groups, positive facial expressions were more frequent and rhythmic motor activities lasted longer with music than with a metronome, and during a live performance rather than a video performance. Relative to patients without CI, patients with CI moved less with music, expressed fewer emotions, and spent less time looking at the musician in the video condition and in the metronome condition. The relative reductions in motor and socio-emotional engagements in patients with CI might be markers of disease progression. However, the presence of a live partner induces older adults to engage emotionally and physically in musical activities emphasizing the relevance of using live performance as motivational levers during music therapy.

## Introduction

In view of the limited effectiveness of drug treatments in the management of patients with dementia, non-pharmacological approaches are increasingly being used to improve the patients’ emotional, social and cognitive functioning and their caregivers’ well-being^1–3^. Music therapy appears to be particularly appropriate, since music is known to modulate the listener’s emotional state and level of motivation^4–6^. Indeed, music generates strong emotions in listeners^7^ and naturally elicits spontaneous motor responses in time with the beat^8,9^. The socio-emotional and motor engagements induced by listening to music might have a key role in the effectiveness of this therapeutic approach^10^.

Music therapy can take various forms: it ranges from individual, passive listening to group music practice (i.e. practice in the presence of therapists, caregivers, other patients, and even musicians). A patient’s socio-emotional reaction to music can be greatly influenced by the social environment. For example, it has been demonstrated that in young adults, reactions such as emotional facial expressions (EFEs) are more frequent in social contexts than in isolation; individuals smile more in response to an event when they are seated near another individual^11,12^. However, it is not known whether this is also true for patients with dementia. Neurodegenerative disease often leads to social withdrawal, which in turn impairs social cognition^13^. More specifically, the abilities of patients with Alzheimer’s disease (AD) to recognize others’ emotions^14^ or to understand others’ mental states^15^ are often impaired. Thus, the influence of the social context on the emotional reactions of patients with neurodegenerative disease is not easy to predict and so must be measured during a musical interaction.

Moreover, a patient’s motor engagement may be sensitive to the presence of others. Studies of young adults have shown that people spontaneously synchronize their movements with those of others. Therefore, observing other people moving to the beat might stimulate patients to move synchronously too^16^. In patients with AD, the literature data show that the motor involvement induced by music is greater in the presence of a musician playing and singing than when the subject is listening to an audio recording of the same piece of music^17–19^. Although this interesting finding highlighted a positive reaction to the presence of a live musician, it is difficult to determine whether this was due to the audiovisual nature of the musical stimulus (compared with an audio recording) or the actual presence of another individual.

In order to distinguish between the effect of sensory stimulation and the effect of a social presence, we recently developed a paradigm for investigating the socio-emotional and motor engagement of patients with moderate-to-severe dementia (due to AD, vascular dementia or mixed dementia) during a musical task^20^. The results revealed that there were no differences in the patients’ EFE production and motor behaviors for a live performance vs. a video performance by the same musician. However, the low frequency of EFEs and rhythmic movements in these patients suggested that their musical engagement was limited—perhaps by the late-stage disease^21,22^. Given that control older adults (i.e. without cognitive impairment (CI)) were not tested in Ghilain et al.’s study, these findings were difficult to interpret and we could not draw firm conclusions about the impact of social context on socio-emotional and motor behaviors during musical activities in patients with dementia.

Patients with dementia appear to remain sensitive to music—even in late-stage disease^23–25^—and are able to spontaneously move or tap with the beat when listening to music^20^. It appears nonetheless that musical pleasure decreases with the severity of CI^21^. Overall, we know that music engages patients with dementia at the emotional and the motor levels, although it not clear whether these behaviors remain intact as the disease progresses.

The objective of the present study was to evaluate the impact of social interaction and musical context on motor and socio-emotional engagement during musical activities in elderly patients with major cognitive impairments (the CI group) and, in comparison, elderly adult participants without CI (the NoCI group). To this end, participants listened to either a musical sequence or a metronomic sequence in front of a musician, in order to assess the effect of the musical context. The musician was either physically present (the live condition) or virtually present through a prerecorded video projected on a 1:1 scale in front of the participant (the video condition). To ensure that participants paid attention to the auditory sequence, they were asked to tap with their hand in time with the beat. The participant’s socio-emotional engagement was measured as the number of positive and negative EFEs (henceforth EFEs^+^ and EFEs^−^, respectively) produced^26^. As demonstrated in the literature, EFEs not only reflect individuals’ emotional states^27^ but are also communication signals. By decoding these EFEs^+^ and EFEs^−^, it is possible to assess the impact of social context on a patient’s socio-emotional engagements^28,29^. Here, we used gaze direction as another potentially valuable marker of social engagement. Indeed, eye contact is a non-verbal behavior that is particularly sensitive to social relationships^30,31^. The participant's motor engagement was assessed in terms of overall body motion^17^ and the numbers of spontaneously produced rhythmic and non–rhythmic movements^18,19^.

According to our hypotheses, the frequency of motor and socio-emotional engagements during musical activities should be higher in the social (live) condition than in the video condition. Since music is associated with motor stimulation and social cohesion^32,33^, non-verbal and rhythmic behaviors should be more frequent in the music condition than in the metronome condition. Lastly, considering the decreased of social cognition with AD, we predicted that socio-emotional engagements would be less frequent in the CI group than in the NoCI group, particularly in video condition.

## Material and methods

### Participants

Ninety-seven participants were recruited at the Bateliers Day Hospital (part of Lille University Medical Center, Lille, France) during a scheduled consultation for memory problems or falls. All participants were right-handed and were native French speakers. The CI group comprised 48 patients suffering from a major Cognitive Impairment, either mild–to–moderate AD (n = 12), mixed dementia (n = 30), or vascular dementia (n = 6). All diagnoses had been made by a geriatrician on the basis of the Diagnostic and Statistical Manual of Mental Disorders—Fifth Edition^34^. The NoCI (control) group comprised 49 matched patients with no signs of CI at the time of testing.

The two groups’ demographic data (included age, sex, educational level^35^, musical expertise^36^), and clinical data (including the State-Trait Anxiety Inventory (STAI)^37^, Activities of Daily Living^38^, and Mini-Mental State Examination^39^ scores) were collected. The study was approved by an investigational review board (*CPP Sud-Est IV*, Lyon, France; reference: 18/012) and registered with the French National Data Protection Commission (*Commission nationale de l'informatique et des libertés* (Paris, France)) and with the ClinicalTrials.gov platform (first posted on the 31/10/2019, identifier NCT04146688). All methods were carried out in accordance with relevant guidelines and regulations. All participants provided their informed, written consent to participation in the study.

### Apparatus

**Figure 1:**
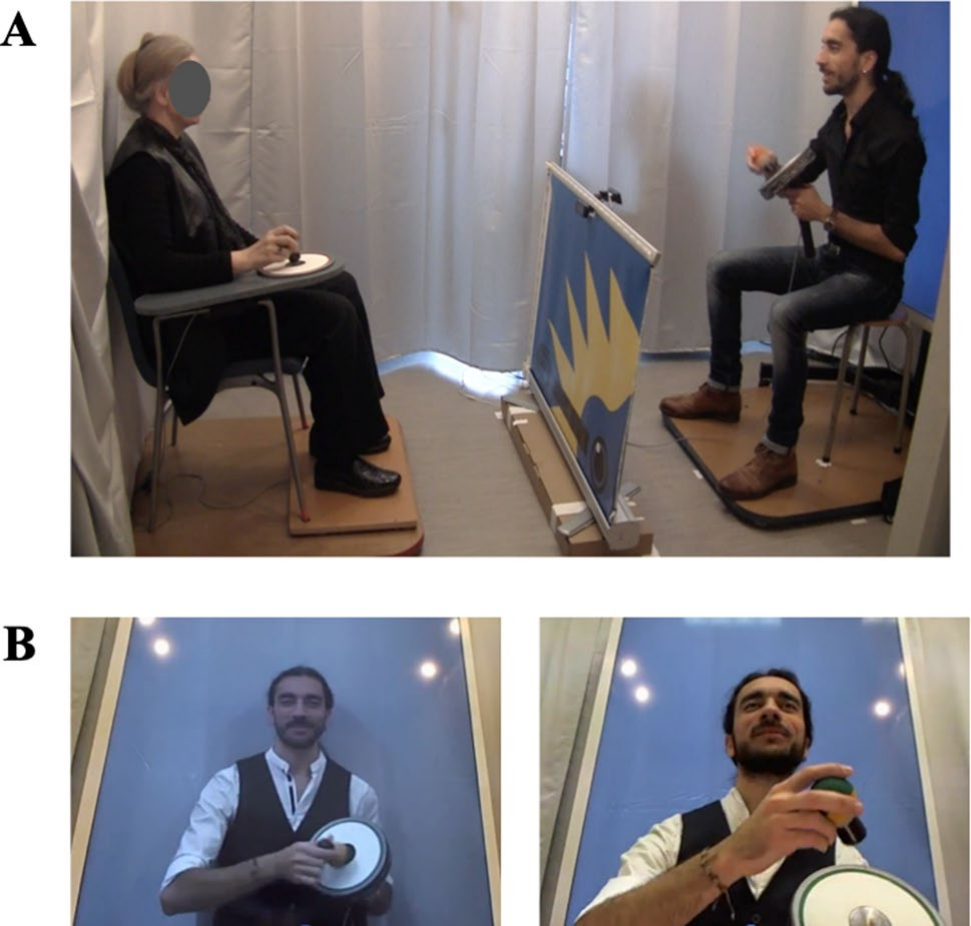
Experimental set-up. (**A**) The participant was seated in front of a musician, who sang and tapped on a tablet in time with the sound sequence’s rhythm. The participant’s chair was placed on force platforms, in order to record his/her overall body motion. (**B**) Manipulation of the social context. The musician’s performance could be displayed on a life-sized screen (the video condition) or truly live (the live condition).

The experimental set-up consisted of a force plate located under the participant's chair, in order to measure his/her overall body motion (Fig. 1, see supplementary material). The participant sat on a chair equipped with a tapping tablet fixed to the right armrest, on which s/he tapped with a tapping device held in their right hand. In the live condition, the musician was seated 200 cm in front of the participant. In the video condition, the musician’s performance was displayed on a 1:1 scale on a 158 × 92 cm screen placed 215 cm from the participant. The participant’s and musician’s behaviors during the tasks were videorecorded.

Sixty-second metronomic and musical sequences were used. Both had a similar tempo (inter-onset interval: 800 ms). The metronomic sequence was composed of regular beats. The musical sequence was an excerpt from a popular 1930s French song ("La Java Bleue") well known to the age group studied here, as attested by post-experiment debriefing (all participants judged the song as very familiar, except the participants who had forgotten that they had been listening to music a few minutes earlier). It has a ternary metric and a strong beat on the first of the metric’s three beats. To give the tempo, four initial beats preceded each sound sequence. The sound stimuli were presented at a comfortable hearing level through two loudspeakers.

### Design and procedure

Each participant was tested individually. The participant was asked to tap on the tablet in time with the beat of the sound sequence. The participant had to perform this tapping task when the musician was seated in front of them; the musician also sang and synchronized his tapping with the sound sequence. For the live condition, the musician was instructed to act as similarly as possible to the prerecorded condition. In each trial, the sound sequence was the metronomic sequence or the musical sequence without lyrics. During the metronomic sequence, the musician pronounced “Ta” with each metronomic beat. During the musical sequence, he sang all the song’s lyrics.

Overall, the experimental design included one between-subjects factor (GROUP, with two levels: CI group/NoCI group), and two within-subjects factors (AUDIO, with two levels: metronomic/musical; and SOCIAL CONTEXT, with two levels: video/live). The four experimental conditions’ order of presentation was counterbalanced.

### Data analysis

We analyzed the participants’ *social and emotional behaviors*, including gaze direction (towards the musician, towards the tapping device, or in another direction), EFEs^+^ (such as joy), and EFEs^−^ (such as sadness, anger, disgust, and fear). The EFEs were coded according to the Facial Action Coding System (Ekman and Friesen, 1976), using Argyle’s method (Argyle, 2013). EFEs^+^ and EFEs^−^ are reported as the frequency (number of behaviors per minute).

We also analyzed the participants’ *spontaneous motor behaviors.* Firstly, the overall body motion (in mV) corresponded to the weight variations measured by the force plate under the participant's chair (Quantity of Motion)^17,40^. Secondly, we measured the time proportion (duration of a given behavior per minute) of spontaneous rhythmic movements of the head and the lips, and the frequency (number of behaviors per minute) of non-rhythmic movements of the head (e.g. turning the head away). Hand movements were not included because tapping behaviors were analyzed in a separate study^41^.

Two independent observers blindly coded the participants’ non-verbal behaviors on the basis of the video recordings alone (i.e. without the audio recordings). Each trial lasted one minute. For each behavior, inter-judge agreement was checked against literature criteria (Harrigan et al.^42^). Computer-assisted coding was performed using Behavioral Observation Research Interactive software^43^. Values obtained for each behavior and in each experimental condition are presented in the Supplementary Material.

### Informed consent

We certify that we have obtained informed consent for the publication of identifying images in an online open-access publication in the methods section (Fig. 1).

## Results

### Preliminary analyses

#### Demographic and clinical data

We compared the CI and NoCI groups with regard to the demographic and clinical data. Data and statistical tests results are depicted in Table 1. There were no statistically significant intergroup differences with regard to age, sex educational level, musical expertise, and the STAI score. In contrast, the two groups differed significantly in the severity of CI (assessed by the MMSE score) and personal independence (as assessed by the ADL score).

**Table 1:** Demographic and clinical characteristics of study participants with or without Major cognitive impairment (the CI and NoCI groups, respectively).

	CI (*N* = 48)	NoCI (*N* = 49)	Test	Result	*p* value
Age	82.8 ± 5.5	80.9 ± 5.2	Studen's *t*-test	t(95) = 1.74	*p* = 0.08
Sex (female/male)	38/10	37/12	Pearson’s chi^2^ test	χ2(1, N = 97) = 0.18	*p* = 0.67
Educational level (4 levels)^a^ ^35^	8/26/6/8	7/19/9/14	Pearson’s chi^2^ test	χ2(3, N = 97) = 3.38	*p* = 0.34
Musical expertise questionnaire (out of 28)^36^	4.3 ± 3.1 ^b^	4.8 ± 3.2	Mann–Whitney U	U = 1075	*p* = 0.46
STAI (out of 40)^37^	29.4 ± 10.8 ^b^	33.2 ± 12.3	Mann–Whitney U	U = 930	*p* = 0.08
ADL (out of 6)^38^	5.0 ± 0.9 ^b^	5.7 ± 0.6	Mann–Whitney U	U = 543	***p* < 0.001**
MMSE (out of 30)^39^	20.2 ± 3.6 ^b^	28.1 ± 1.2	Mann–Whitney U	U = 0	***p* < 0.001**

Data are quoted as the mean ± standard deviation (SD).

*STAI* state-trait anxiety inventory, *ADL* activities of daily living, *MMSE* mini mental state examination.

^a^Educational level. Level 1: primary education only; Level 2: junior high diploma; Level 3: high school diploma; Level 4: college diploma.

^b^Missing data for one participant.

The *p*-values of significant statistical tests are in bold.

### Socio-emotional behaviors

#### Positive emotional facial expressions

**Figure 2:**
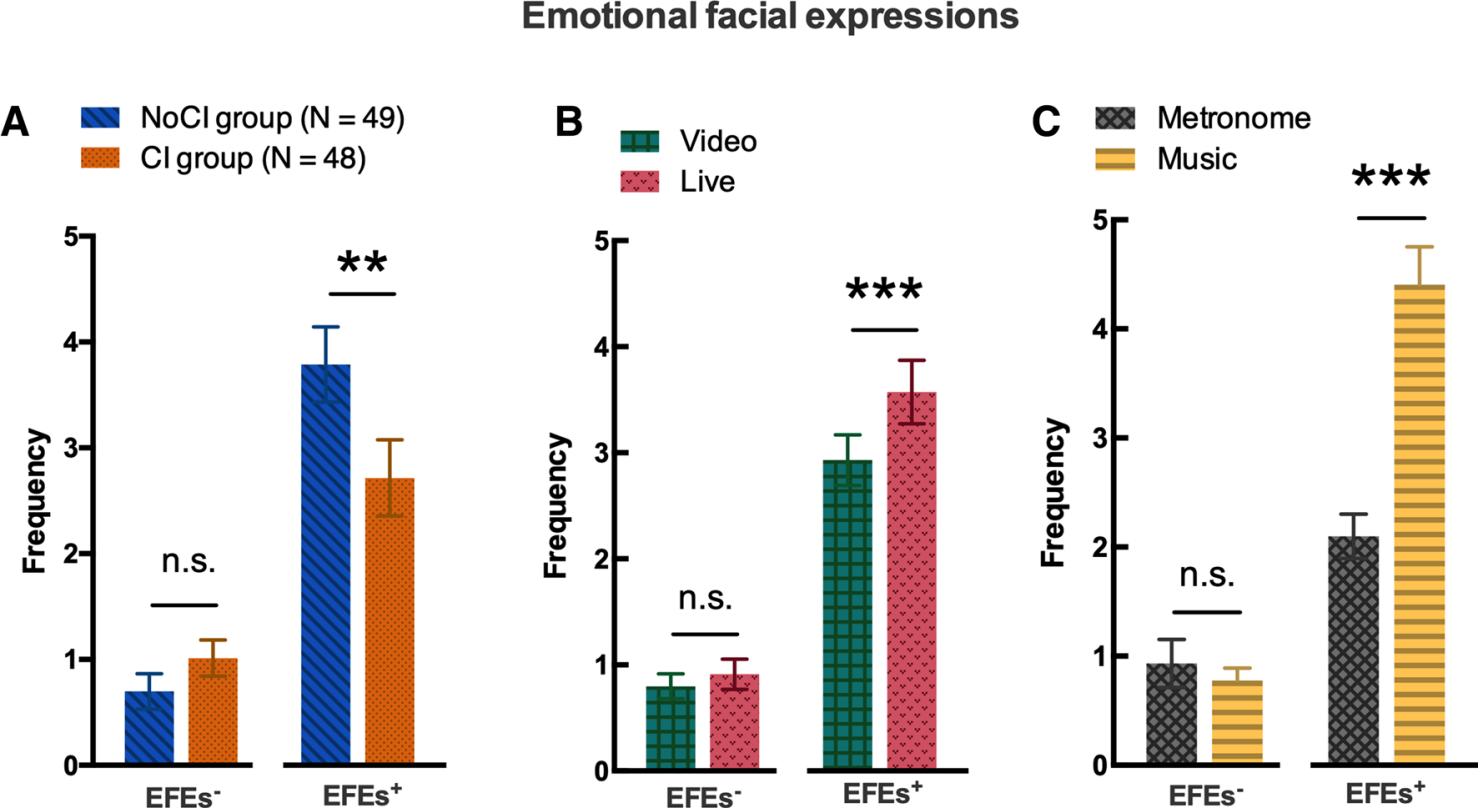
Emotional facial expressions. The mean ± SEM frequency of EFEs^+^ and EFEs^−^, as a function of (**A**) the group (CI/NoCI), (**B**) the social context (video or live performance), and (**C**) the sound sequence (metronome or music). The production of EFEs^−^ was not influenced by the group or any of the experimental conditions. In contrast, EFEs^+^ were more frequent in the NoCI group than in the CI group. Moreover, EFEs^+^ were more frequent in the live condition than in the video condition and more frequent in the musical condition than in the metronome condition.

A three-way analysis of variance (ANOVA) was conducted on the frequency of the positive EFEs with the between-subjects factor GROUP (two levels: CI/NoCI), and the within-subjects factors AUDIO (two levels: metronomic/musical) and SOCIAL CONTEXT (two levels: video/live). The analysis revealed a significant effect of the GROUP (F_(1,95)_ = 4.48, *p* < 0.05, η^2^_p_ = 0.045). Participants in the CI group expressed fewer EFEs^+^ than participants in the NoCI group (see Fig. 2A). The analysis also showed a significant effect of the SOCIAL CONTEXT (F_(1,95)_ = 11.2, *p* < 0.01, η^2^_p_ = 0.105). Participants expressed more EFEs^+^ in the live condition than the video condition (see Fig. 2B). Lastly, we found a significant effect of the AUDIO (F_(1,95)_ = 79.0, *p* < 0.001, η^2^_p_ = 0.454). The participants expressed more EFEs^+^ while listening to music than while listening to the metronome (see Fig. 2C). There were no other interactions.

#### Negative emotional facial expressions

A three-way analysis of variance (ANOVA) was conducted on the frequency of the negative EFEs with the between-subjects factor GROUP (two levels: CI/NoCI), and the within-subjects factors AUDIO (two levels: metronomic/musical) and SOCIAL CONTEXT (two levels: video/live) (see Fig. 2). The analysis revealed no significant interactions or main effects.

#### Gaze direction

We first evaluated the time proportions of gaze directed in three directions: towards the musician, towards the tablet, or towards other parts of the environment. To this end, we conducted a one-way ANOVA on the time proportion of gaze direction with the between-subjects factor DIRECTION (three levels: Musician/Tablet/Other). Our analysis revealed a significant effect of DIRECTION (F_(1,96)_ = 66.57, *p* < 0.001, η_p_^2^ = 0.409). Participants spent more time looking at the musician (mean ± SEM: 0.44 ± 0.03) and at the tablet (mean ± SEM: 0.45 ± 0.03) than at other parts of the environment (mean ± SEM: 0.03 ± 0.01; Fisher’s post-hoc tests: *p* < 0.001). Considering that the participants spent about 97% of their time looking at the musician or at the tablet, we excluded the 3% of the time during which participants looked in other directions from the subsequent analysis.

**Figure 3:**
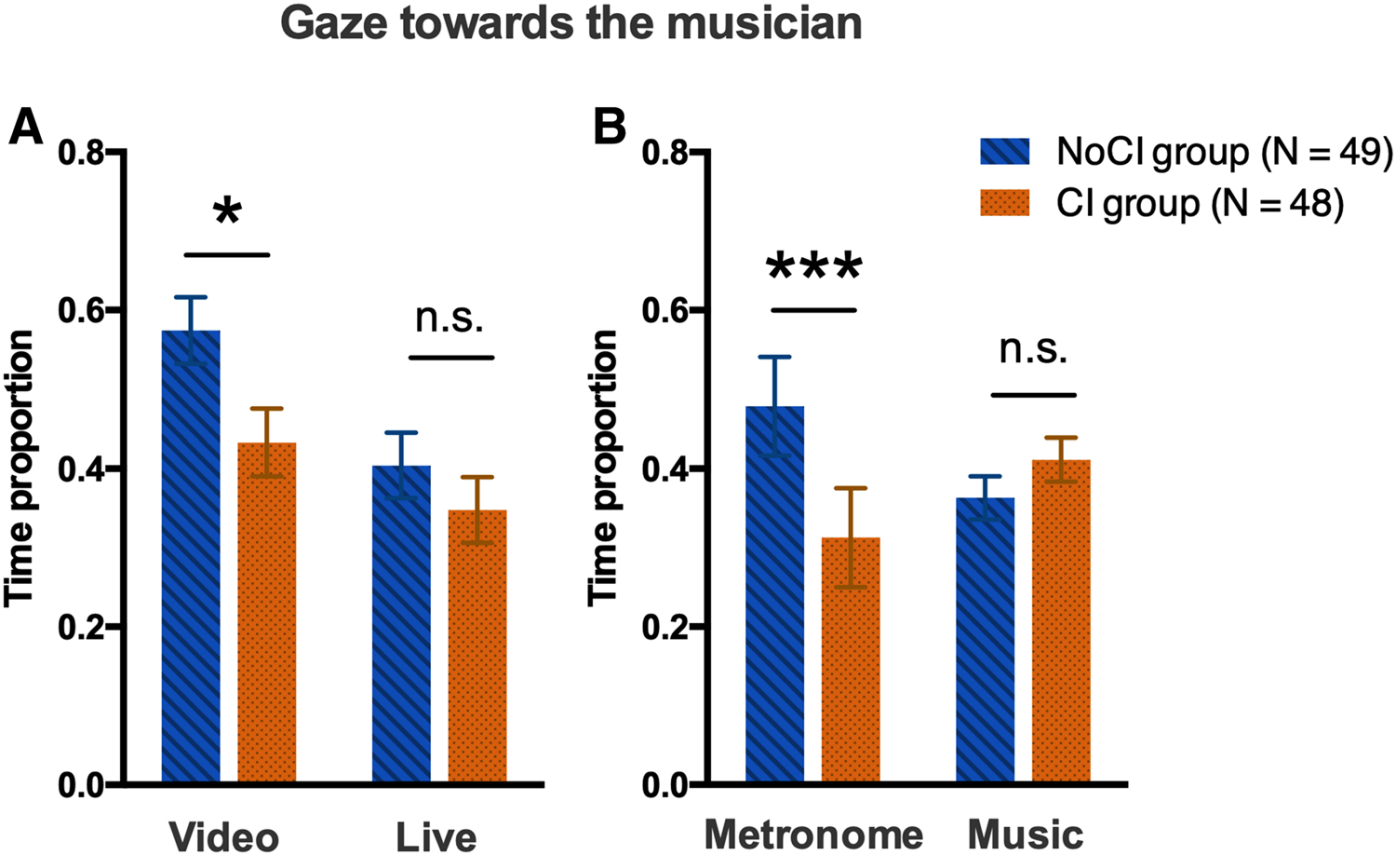
Gaze towards the musician. The mean ± SEM time proportion of gaze towards the musician, as a function of the group (CI/NoCI) and (**A**) the social context (video or live) and (**B**) the sound sequence (metronome or music). Participants in the CI group looked at the musician for less time than participants in the NoCI group in both the video conditions and the metronome conditions. There were no intergroup differences in the live or music conditions.

We next analyzed only the time proportion of gaze towards the musician. We considered that if the participants were not looking at the musician, they were looking at the tablet. A three-way ANOVA was conducted on the time proportion of gaze towards the musician with the between-subjects factor GROUP (two levels: CI/NoCI) and the within-subjects factors AUDIO (two levels: metronomic/musical) and SOCIAL CONTEXT (two levels: video/live). The analysis revealed a significant interaction between GROUP and SOCIAL CONTEXT (F_(1,95)_ = 4.61, *p* < 0.05, η^2^_p_ = 0.046). In the video condition, participants in the CI group looked less at the musician than participants in the NoCI group did (Fisher’s post-hoc test: *p* < 0.05). In the live condition, the time proportion of gaze towards the musician did not depend on the group (see Fig. 3A). We also obtained a significant interaction between GROUP and AUDIO on the proportion of gaze towards the musician (F_(1,95)_ = 5.30, *p* < 0.05, η^2^_p_ = 0.053). When listening to a metronome, participants in the CI group looked less at the musician than participants in the NoCI group did (Fisher’s post-hoc test: *p* < 0.001). In the music condition, the time proportion of gaze towards the musician did not depend on the group (see Fig. 3B). Lastly, the results showed significant main effects of SOCIAL CONTEXT (F_(1,95)_ = 40.74, *p* < 0.001, η^2^_p_ = 0.300), and AUDIO (F_(1,95)_ = 9.11, *p* < 0.01, η^2^_p_ = 0.0.87). There were no effects of GROUP and no other interactions.

### Spontaneous motor behaviors

#### Overall body motion

**Figure 4:**
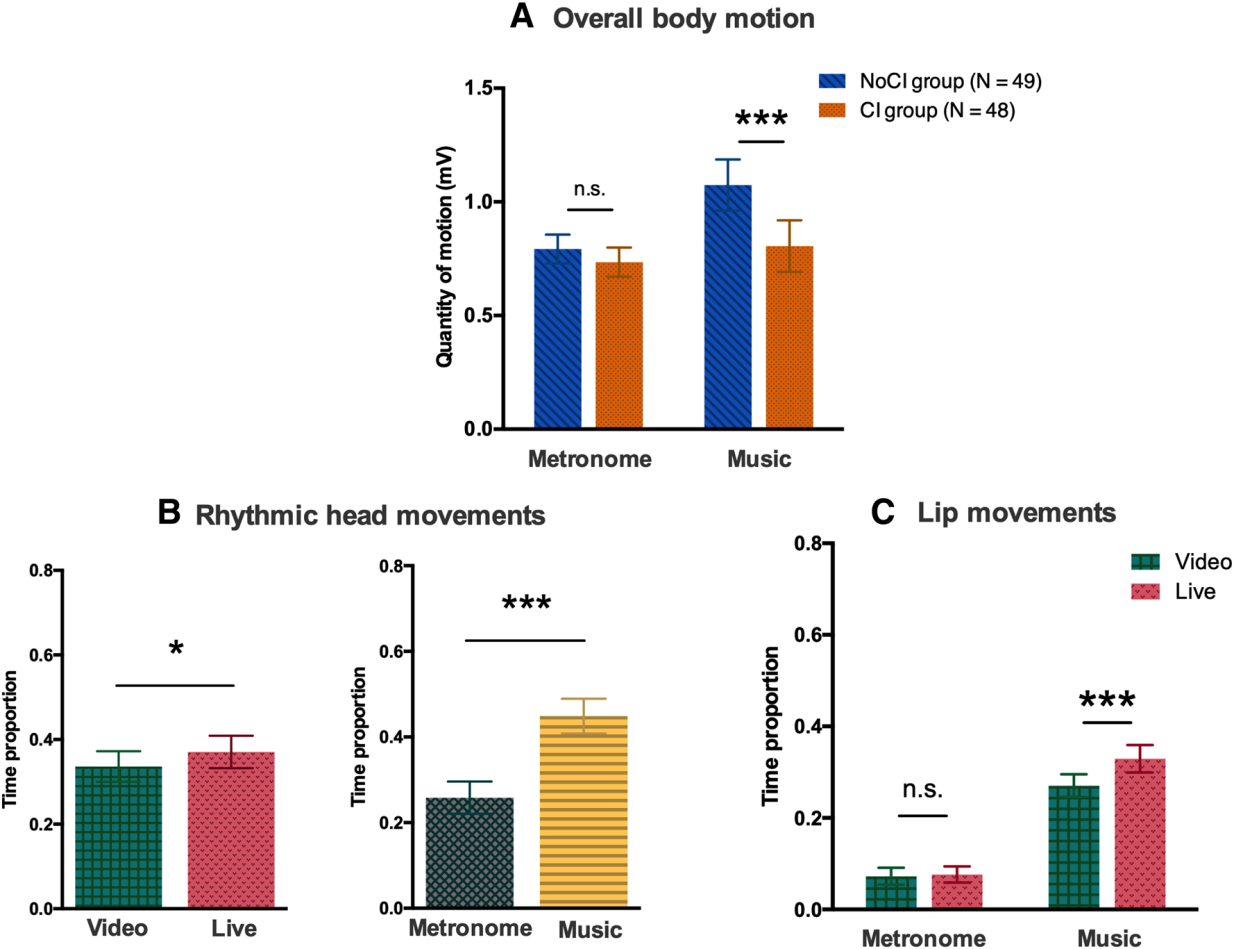
Spontaneous motor behaviors. (**A**) Mean ± standard error of the mean (SEM) overall body motion as a function of the sound sequence (metronome or music) and the group (CI/NoCI). In the music condition, participants in the CI group produced less overall body motion than participants in the NoCI group. The NoCI group producing more overall body motion when listening to the musical sequence than when listening to a metronome. In contrast, overall body motion in the CI group did not depend on the sound sequence. (**B**) Mean ± SEM time proportion of rhythmic head movements, as a function of the social context (video or live) and the sound sequence (metronome or music). Participants produced more frequent rhythmic head movements in the live condition than in the video condition (left graph) and more when listening to music than when listening to the metronome sequence (right graph). (**C**) Mean ± SEM time proportion of lip movements, as a function of the social context (video or live) and the sound sequence (metronome or music). Participants moved their lips less frequently when listening to the metronome than when listening to music. With music, participants moved their lips more frequently in the live condition than in the video condition.

A three-way ANOVA was conducted on the overall body motion, with the between-subjects factor GROUP (two levels: CI group/NoCI group), and the within-subjects factors AUDIO (2 levels: metronome/music) and SOCIAL CONTEXT (2 levels: video/live). The analysis revealed a significant interaction between GROUP and AUDIO (F_(1,95)_ = 3.95, *p* < 0.05, η^2^_p_ = 0.040) (see Fig. 4A). When listening to music, participants in the NoCI group produced more spontaneous body motion than the participants in the CI group did (Fisher’s post-hoc test: *p* < 0.001). In the metronome condition, there was no intergroup difference in overall body motion. Moreover, participants in the NoCI group produced more body motion when listening to music than when listening to the metronome (Fisher’s post-hoc test: *p* < 0.01). We also observed a significant main effect of the AUDIO (F_(1,95)_ = 10.97, *p* < 0.01, η^2^_p_ = 0.10). There were no other main effects or interactions.

#### Rhythmic head movements

A three-way ANOVA was conducted on the time proportion of rhythmic head movements, with the between-subjects factor GROUP (two levels: CI/NoCI) and the within-subjects factors AUDIO (two levels: metronomic/musical) and SOCIAL CONTEXT (two levels: video/live). The analysis revealed a significant main effect of the SOCIAL CONTEXT (F_(1,95)_ = 4.18, *p* < 0.05, η^2^_p_ = 0.042). Participants made more rhythmic head movements in the live condition than in the video condition (Fisher’s post-hoc test: *p* < 0.05; Fig. 4B). There was also a significant main effect of the AUDIO (F_(1,95)_ = 61.60, *p* < 0.001, η^2^_p_ = 0.39). Participants made more rhythmic head movements when listening to music than when listening to a metronome (post-hoc Fisher’s test: *p* < 0.001). There were no effects of GROUP and no interactions.

#### Lip movements

A three-way ANOVA was conducted on the time proportion of lip movements with the between-subjects factor GROUP (two levels: CI/NoCI) and the within-subjects factor AUDIO (two levels: metronomic/musical) and SOCIAL CONTEXT (two levels: video/live). The analysis revealed a significant interaction between the factors AUDIO and SOCIAL CONTEXT (F_(1,95)_ = 6.15, *p* < 0.05, η^2^_p_ = 0.061; Fig. 4C). Participants sang more when listening to music than when listening to a metronome. When listening to music, participants sang more in the live condition than in the prerecorded condition (Fisher’s post-hoc test: *p* < 0.001). When listening to a metronome, SOCIAL CONTEXT did not impact the participants’ singing time. The results also showed significant main effects of AUDIO (F_(1,95)_ = 70.09, *p* < 0.001, η^2^_p_ = 0.43), and SOCIAL CONTEXT (F_(1,95)_ = 8.73, *p* < 0.01, η^2^_p_ = 0.084). There was no effect of GROUP effect or any other interactions.

#### Non-rhythmic head movements

A three-way ANOVA on the time proportion of non-rhythmic head movements with the between-subjects factor GROUP (two levels: CI/NoCI) and the within-subjects factor AUDIO (two levels: metronomic/musical) and SOCIAL CONTEXT (two levels: video/live) did not show any main effects or significant interactions.

## Discussion

The objective of the present study was to evaluate the respective impacts of social presence and auditory context on the socio-emotional and motor engagement of elderly patients with major CI vs. matched participants without CI during musical activities. In line with our expectations, the participants’ engagements varied with the social and auditory conditions. The EFEs^+^ were more frequent and the rhythmic head movements lasted longer in the presence of a live musician than in the presence of a virtual musician. Likewise, the EFEs^+^ were more frequent and the rhythmic head movements lasted longer during music than during metronome sequences. Participants spent more time producing lip movements when listening to a live song than when listening a pre-recorded (video) song. Furthermore, the participants’ motor and socio-emotional behaviors were influenced by CI. Patients with major CI moved less with music, expressed fewer EFEs^+^, and spent less time looking at the musician in the video condition and in the metronome condition than participants with NoCI.

Our study’s major finding was that the participants’ socio-emotional and motor engagement in musical activities depended on the social context. Participants produced more EFEs^+^ when facing a live partner than when facing a video recording. Hence, our study of the effect of the social context on positive emotional reactions extends the results observed in young adults^11,12,44^ to elderly adults with major CI. When facing a live singer, participants with major CI produced more rhythmic head movements and lip movements—suggesting that they were trying to sing along. This finding is in line with literature data on patients with CI showing that the presence of another person increases motor engagement with music^17,18^. Taken as a whole, these results strongly suggest that the actual presence of a musician or singer induces elderly adults to engage emotionally and physically in musical activities. This social effect might be a valuable lever for involving patients with CI in music therapy and increasing the latter’s benefits.

In agreement with our predictions, EFEs^+^ were more frequent during musical sequences than during metronome sequences. These results confirm that music can still induce positive emotions in elderly listeners with dementia, as reported in the literature^19^. As expected, participants’ motor behaviors (lip movements and rhythmic head movements) were more frequent when listening to music than when listening to the metronome. In line with our previous study^20^, these results suggest that elderly adults with or without CI are still sensitive to musical rhythm, which encourages them to move with the beat and sing along with the song^17,18^. The lack of an effect on the frequency of non-rhythmic movements confirms that music promotes rhythmic movements specifically and not motor behaviors in general.

Interestingly, the results of the present study suggest that the socio-emotional and motor engagement of participants differed according to the presence or absence of CI. EFEs^+^ were less frequent in the CI group than in the NoCI group—as would typically be expected with the development of apathy and depression in people with dementia^45^. This finding might be linked to a decrease in musical pleasure^21^. Moreover, the difference in overall body motion between the CI and NoCI groups was observed for music sequences but not for metronome sequences. In other words, listening to music clearly improved the motor engagement in the NoCI group but not in the CI group. Even though patients with major CI still moved with the musical rhythm (as evidenced by the increase in rhythmic head movements), their overall body motion was less ample than in participants without CI. The decrease in motor and socio-emotional engagement in elderly patients with major CI should be examined in detail, since it might constitute (1) a diagnostic marker of neurodegenerative disease progression and (2) a prognostic marker of the effectiveness of music therapy.

Lastly, we found a social effect on gaze direction: participants diverted their gaze from the musician more in the live condition than in the video condition. The sole presence of the live musician may create embarrassment or social pressure^46^ to which participants react by averting their gaze. Moreover, our results indicated that the presence of CI influences gaze direction. Relative to participants without CI, patients with major CI looked less towards the musician (and so more towards the tablet or their hands) in both the video condition and the metronome condition. There are two possible main explanations for this finding. Firstly, looking at the hand might improve motor control, which can be useful in tapping tasks. However, the experimental conditions under which patients with major CI looked at their hands more than participants with NoCI did (i.e. tapping in time with a metronome) were not the most challenging ones (the most challenging being tapping with music^47^, as attested results in a tapping task in patients with cognitive impairments^20,41^). Secondly, averting the gaze from the musician might be a sign of anxiety or social pressure during the task. Patients with major CI diverted their gaze from the musician more than controls during the more artificial, less stimulating conditions (i.e. the video conditions and the metronome conditions). We therefore hypothesize that patients with major CI may be more sensitive to anxiety or social pressure and therefore diverted their gaze in what was a relatively stressful, unusual condition. In contrast, they acted in much the same way as the matched participants with NoCI when listening to live music—perhaps because moving to the rhythm with another individual present is a pleasant, ecological social task and is similar to a real-life situation^32,48^. Studying modifications of social cognition in patients with dementia might help to understand the effects of CI on gaze direction observed here.

In conclusion, our study is the first to evidence that even though patients with mild-to-moderate dementia still react positively to music and move with the rhythm, they engage less than participants without CI. This novel result shows that while musical cognition is thought to be relatively unaffected by neurodegenerative disease, it may nevertheless decrease with disease progression. This novel result needs to be interpreted with caution, as we cannot exclude that others differences between the groups may explain those results. Reactions to music should be studied in more detail because they might be diagnostic markers of disease progression but also prognostic markers of the effectiveness of music therapy. Our results also indicate that a live social context accentuates socio-emotional and motor engagement with music by older adults with or without CI. This novel finding emphasizes the relevance of using live performance and social interaction as motivational levers during music therapy.

## Supplementary Information

Supplementary Information 1.

Supplementary Video 1.

Supplementary Video 2.

Supplementary Video 3.

Supplementary Video 4.
