# Supplementary material for: Socio-emotional and motor engagement during musical activities in older adults with major neurocognitive impairment
Source: Sci Rep. 2021 Jul 27;11:15291. doi: 10.1038/s41598-021-94686-4 (PMC8316394; doi:10.1038/s41598-021-94686-4)
Supplement: Supplementary file 1 — Supplementary Information 1. [file 41598_2021_94686_MOESM1_ESM.pdf]

SUPPLEMENTARY INFORMATION

Socio-emotional and motor engagement during musical activities in cognitively impaired older adults

Lise Hobeika[1,2*], Matthieu Ghilain[1*], Loris Schiaratura[1], Micheline Lesaffre[3], Dominique Huvent-Grelle[4], François Puisieux[4] and Séverine Samson[1,5]

[1] Université de Lille, ULR 4072 – PSITEC – Psychologie: Interactions, Temps, Emotions, Cognition, F-59000 Lille, France
[2] Sciences et Technologies de la Musique et du Son, IRCAM, CNRS, Sorbonne Université, F-75004 Paris, France
[3] IPEM, Department of Art History, Musicology and Theatre Studies, Ghent University, Belgium
[4] Hôpital Gériatrique les Bateliers, Pôle de Gérontologie, CHU Lille, 23 rue des Bateliers, F-59037 Lille France
[5] AP-HP, GHU Pitié-Salpêtrière-Charles Foix, F-75013 Paris, France

I. Values obtained for each behavior and in each experimental condition are presented here.

A. Socio-emotional behaviors

			Metronome		Music	
			**Video**	**Live**	**Video**	**Live**
**EFE+**	**CI group**	*Mean ± SEM*	1.45 ± .26	1.68 ± .24	3.64 ± .49	4.09 ± .56
	**NoCIgroup**	*Mean ± SEM*	2.34 ± .32	2.91 ± .44	4.29 ± .48	5.60 ± .61
**EFE-**	**CI group**	*Mean ± SEM*	.85 ± .31	1.10 ± .47	.98 ± .20	1.12 ± .23
	**NoCIgroup**	*Mean ± SEM*	.94 ± .28	.84 ± .20	.43 ± .15	.59 ± .16
**Gaze towards the musician**	**CI group**	*Mean ± SEM*	.36 ± .05	.67 ± .07	.51 ± .05	.43 ± .05
	**NoCIgroup**	*Mean ± SEM*	.57 ± .05	.43 ± .07	.58 ± .04	.42 ± .05

2. Spontaneous motor behaviors

			Metronome		Music	
			**Video**	**Live**	**Video**	**Live**
**Quantity of Motion**	**CI group**	*Mean ± SEM*	.75 ± .05	.72 ± .05	.80 ± .06	.81 ± .05
	**NoCIgroup**	*Mean ± SEM*	.80 ± .05	.79 ± .04	.95 ± .06	1.20 ± .19
**Rhythmic movements of the Head**	**CI group**	*Mean ± SEM*	.25 ± .04	.28 ± .04	.43 ± .04	.42 ± .04
	**NoCIgroup**	*Mean ± SEM*	.22 ± .04	.27 ± .04	.44 ± .04	.51 ± .04
**Rhythmic movements of the Lips**	**CI group**	*Mean ± SEM*	.05 ± .02	.03 ± .01	.27 ± .04	.33 ± .04
	**NoCIgroup**	*Mean ± SEM*	.10 ± .03	.12 ± .03	.27 ± .03	.33 ± .04
**Non-Rhythmic movements**	**CI group**	*Mean ± SEM*	4.86 ± .61	4.22 ± .46	5.59 ± .77	5.17 ± .73
	**NoCIgroup**	*Mean ± SEM*	4.96 ± .79	4.91 ± .71	4.26 ± .70	3.39 ± .53

II. Inter-judges agreements for each decoded behavior

		r	*p*
**Socio-emotional behaviors**	EFE+	.95	< .001
	EFE-	.91	< .001
	Gaze toward the musician	.97	< .001
	Gaze towards the tablet	.89	< .001
	Other gaze directions *	-	-
**Spontaneous rhythmic motor behaviors**	Head	.79	.002
	Lips	.98	< .001
**Non-rythmic behaviors**	Head	.75	.005

* The percentage of gaze oriented towards other directions than the musician and the tablet were not frequent enough to allow a statistical analysis of inter-judge reliability
